# Post-Septoplasty Palatal Fistula in A Patient with Normal Palate: Case Report

**DOI:** 10.29252/wjps.7.3.382

**Published:** 2018-09

**Authors:** Gholamreza Motazedian, Ali Akbar Mohammadi, Soheil Mohammadi

**Affiliations:** 1 Burn and Wound Healing Research Center, Plastic and Reconstructive Surgery Ward, Shiraz University of Medical Sciences, Shiraz, Iran;; 2 Faculty of Medicine, Tehran University of Medical Sciences, Tehran, Iran

**Keywords:** Septoplasty, Palatal Fistula, Iran

## Abstract

Nasal septoplasty is a common procedure performed in plastic surgery and otorhinolaryngology. Many complications after septoplasty have been reported. Palatal perforation is one of the rarest complications with only a few cases reported in the literature. The reported cases had misdiagnosed submucousal cleft palate or high arched palate, but a patient with palatal perforation after septoplasty is presented here which have had neither evidence of submucousal cleft palate nor high arched palate.

## INTRODUCTION

Causes of palatal fistula are developmental, infectious, autoimmune, drug related, iatrogenic and many other rare causes such as rhinolithian. Nasal septoplasty is a common and low-risk procedure being performed in plastic surgery and otorhinolaryngology. Many complications after septoplasty have been reported. The most common complications are bleeding, adhesions, septal hematoma, saddle nose deformity and nasal septal perforation. Palatal perforation is one of the rarest complications. A few cases have been reported in the literature which regardless of their size and location they have various symptoms such as nasal regurgitation and nasal emission. The reported cases had misdiagnosed submucousal cleft palate or high arched palate.^1^^-^^3^ Here, we present a patient with palatal perforation after septoplasty which had no submucousal cleft palate or high arched palate.

## CASE REPORT

**Fig. 1:**
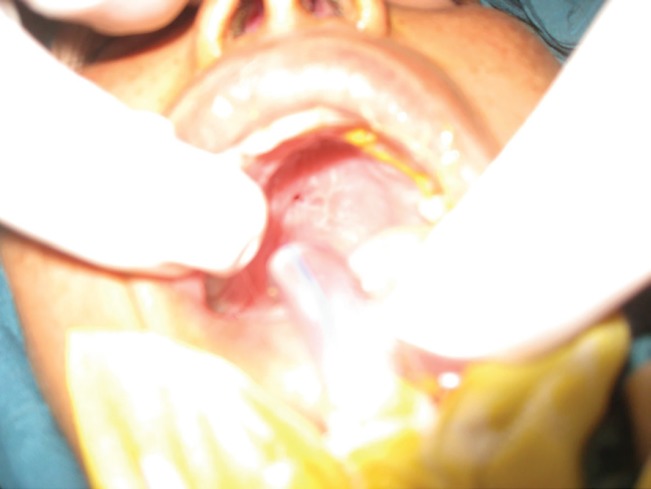
Post-septoplasty palatal fistula in an otherwise normal palate of a 31 y/o female patient

**Fig. 2:**
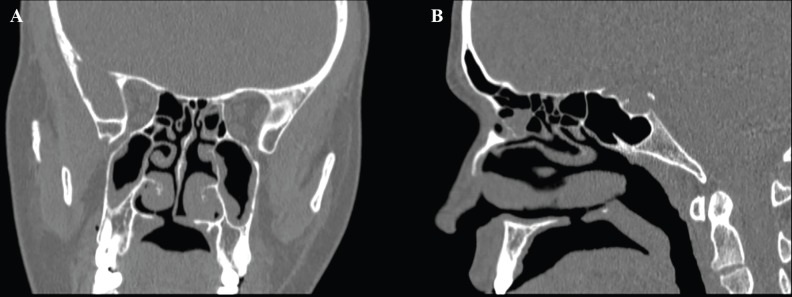
Palatal CT scan showing palatal fistula in an otherwise normal palate of the patient; Palatal arch is normal; coronal view (a), axial view (b)

A 31 year-old female patient referred to our clinic due to a palatal perforation secondary to septorhinoplasty performed 2 years before, with minimal regurgitation and hypernasal speech. The patient had history of 2 failed fistula closure operations. On examination, a small perforation in the hard palate was observed (Figure 1). There was not any undiagnosed underlying submucousal cleft palate or high palatine vault (Figure 2). She was subjected to surgery under general anesthesia. Palatal perforation was repaired with mucosal hinge flap as nasal lining and a mucoperiosteal rotational flap for oral coverage. In the subsequent follow-up, no recurrence of fistula was observed and the problems of regurgitation and hypernasal speech were solved.

## DISCUSSION

Following septoplasty, many complications such as breathing difficulty, toxic shock syndrome, hemorrhage, vestibulitis, hematoma, adhesions, septal hematoma, septal perforation , etc. were reported.^1^^-^^3^ Palatal perforation is a very rare complication and literature review shows very few reported cases.^4^ Ersoy *et al. *reported a case of post-septoplasty palatal fistula that the diagnosis of submucous cleft palate as the underlying cause was made after septoplasty.^4^ Gökdemir and Bal reported a case similar to our case but, during the examination of the patient they figured out that his palatine vault was high and its concavity was more than normal.^5^

Balmor *et al.* also reported a case of the renal cell carcinoma, receiving biophosphanate treatment. The patient had sleep apnea and recurrent bleeding due to nasal polyp. Nasal surgery was performed for a nasal polyp and the patient developed palatal perforation after the surgery. It was postulated that the palatal fistula was caused by the osteonecrosis which developed due to the biophosphanate treatment.^6^ In the literature, it was emphasized that the local flaps could be sufficient for closure in small fistula, while in larger defects, a multidisciplinary approach should be used.^7^

In our case, the palatal perforation developed as a complication after the septal surgery in an otherwise normal palate. In contrary to previous case reports no submucousal cleft palate or high arched palate were observed on midface CT scan of our patient. Aggressive and incautious osteotomy of septal bony part (vomer and ethmoidal plate) resulted in this complication. To prevent such complications, we suggest to perform a precise and detailed examination prior to surgery to avoid such complications. Also, surgeons should be cautious in excising the bony part of septum and avoid uncontrolled osteotomy in all septoplasty patients, especially in high arched palate and submucousal cleft palate patients. Palatal perforation is a rare complication of septoplasty. Precise pre-operative examination of the patient and cautious gradual osteotomy under direct vision are key points to avoid this annoying complication.

## CONFLICT OF INTEREST

The authors declare no conflict of interest.
